# In this month’s Bulletin

**DOI:** 10.2471/BLT.24.001024

**Published:** 2024-10-01

In the editorial section, Lisa Askie et al. (682) introduce this special issue on the impact of the World Health Organization’s (WHO) normative and standard-setting functions. Catherine Regis et al. (683) examine the challenges of impact evaluation of WHO’s normative output. Elizabeth F Peacocke et al. (684) describe the value proposition of WHO’s *Model list of essential medicines*.

Gary Humphreys (687–688) reports on efforts to adapt guidance on the management of medical and surgical emergencies in hospitals. Julie Makani talks to Gary Humphreys (689–690) about the evolution of treatment guidelines for sickle cell disease.

## Australia 

### Food label policies: voluntary or mandatory?

Alexandra Jones et al. (691–698) observe differential uptake.

Lindsey Smith Taillie and Ana Clara Duran (765–768) make the case for mandatory policies.

## Caribbean countries and territories 

## Germany

### Making COVID-19 guidelines fit-for-context

Tracy Evans-Gilbert et al. (699–706) report steps of local adaptation through the public health agency.

Eva A Rehfuess et al. (742–748) use the WHO-INTEGRATE framework for school adaptation.

## Indonesia 

### Preventing violence against women

Alegra Wolter et al. (730–735) document adaptation of WHO’s RESPECT framework.

## Malawi, Nigeria and South Africa

### Increasing the use of child health guidelines 

Tamara Kredo et al. (749–756) build local capacity. 

## Solomon Islands

### Increasing immunization coverage

Reta Angessa et al. (736–741) report using the reach-every-district approach.

## WHO European Region 

### National guideline adaptations

Marge Reinap et al. (715–721) work with European countries to improve contextualisation of WHO guidelines.

## Global

### Medical products of human origin

Eoin McGrath et al. (707–714) argue for improved global standards.

### WHO *Model list of essential medicines*

Thomas Piggott et al. (722–729) propose future use-cases.

### Living evidence syntheses 

Samantha Chakraborty et al. (757–759) explore implications for health policy.

### WHO guideline development

Manjulaa Narasimhan et al. (760–764) detail community engagement examples.

